# Observing the interplay between surface and bulk optical nonlinearities in thin van der Waals crystals

**DOI:** 10.1038/srep22620

**Published:** 2016-03-03

**Authors:** Skylar Deckoff-Jones, Jingjing Zhang, Christopher E. Petoukhoff, Michael K.L. Man, Sidong Lei, Robert Vajtai, Pulickel M. Ajayan, Diyar Talbayev, Julien Madéo, Keshav M. Dani

**Affiliations:** 1 Femtosecond Spectroscopy Unit, Okinawa Institute of Science and Technology Graduate University, 1919-1 Tancha, Onna, Japan; 2 Department of Materials Science and NanoEngineering, Rice University, Houston, Texas, 77005, USA; 3 Department of Physics and Engineering Physics, Tulane University, 6400 Freret Street, New Orleans, Louisiana 70118, US

## Abstract

Van der Waals materials, existing in a range of thicknesses from monolayer to bulk, allow for interplay between surface and bulk nonlinearities, which otherwise dominate only at atomically-thin or bulk extremes, respectively. Here, we observe an unexpected peak in intensity of the generated second harmonic signal versus the thickness of Indium Selenide crystals, in contrast to the quadratic increase expected from thin crystals. We explain this by interference effects between surface and bulk nonlinearities, which offer a new handle on engineering the nonlinear optical response of 2D materials and their heterostructures.

Over the past decade, van der Waals crystals have provided a rich playground in the exploration of physical phenomena through the transition from three-dimensional bulk crystals to two-dimensional atomically thin layers. For example, monolayers of graphene exhibit Dirac quasiparticles with a relativistic dispersion, in contrast to the electronic properties of bulk graphite^1^. Similarly, in regard to optical phenomena, transition metal dichalcogenides (TMDCs) like MoS_2_ exhibit striking photoluminescence due to the appearance of a direct bandgap for monolayers, as opposed to the indirect bandgap in few-layer or bulk crystals^2^,^3^. Beyond these novel electronic and linear optical properties, the exploration of nonlinear optical phenomena in van der Waals crystals also provides promising possibilities. The changing electronic structure^1^,^2^,^3^,^4^, crystal symmetry^5^, and dimensionality of van der Waals crystals with decreasing numbers of layers impacts the nonlinear response, which depends sensitively on these parameters^6^,^7^,^8^,^9^. Nonlinear optical probes themselves are well suited to exploring electronic and crystal structures of surfaces^7^ and atomically thin materials^10^, such as time-reversal symmetry properties in topological insulators^11^,^12^ and real time monitoring of surface phase transformations in silicon^8^,^13^. Atomically thin materials also have potential applications in chip-scale nonlinear devices^14^,^15^ with electrically tunable capabilities^16^, and as non-invasive probes of charge and current distributions in low dimensional electronic devices^17^.

Exploration of nonlinear optical properties in van der Waals crystals has recently begun, with demonstrations of phenomena unique to these materials. For example, in TMDCs, owing to broken inversion symmetry in just the monolayer, one observes the emergence of strong second harmonic generation (SHG), despite the absence of a nonlinear response in the centro-symmetric bulk crystal^5^,^18^,^19^,^20^. In monolayer WSe_2_, electric fields allow tunability and control of the second-order optical nonlinearities via charge-induced SHG^17^ and control over excitonic oscillator strengths^16^. Similarly, MoS_2_ 21 and Bi2Se_3_ 22 exhibit plasmon-related enhancements in their second-order nonlinearities. In other demonstrations, monolayer GaSe crystals have been reported to exhibit the highest nonlinear susceptibilities among 2D materials to date, as well as compared to standard efficient bulk nonlinear crystals^23^. Further, atomically thin layers of GaSe have been shown to exhibit the well-known quadratic dependence of SHG intensity on thickness, expected for very thin crystals where phase matching considerations are not relevant^24^. InSe, a close cousin of GaSe in the III-VI family of van der Waals crystals, also exhibits large optical nonlinearities in bulk^25^. It has the further advantage of being more stable under ambient conditions in atomically thin form^26^, and is expected to outperform other III-VI van der Waals materials in opto-electronic devices in the visible^27^. Given the range of potential thicknesses, van der Waals crystals like InSe, which maintain broken inversion symmetry for all layer thicknesses, provide another interesting opportunity. They allow exploration of the interplay between surface and bulk contributions to the nonlinear signal, which otherwise dominate only at the atomic and bulk extremes, respectively. Previously, such an interplay between strong bulk and weak surface nonlinearities has led to unique nonlinear symmetries in bulk GaAs^28^. In van der Waals crystals, by controlling the thickness layer-by-layer, one obtains fine control over relative strengths of bulk and surface contributions and thus explore interesting aspects of the transition of nonlinear optical phenomena from 2D to 3D. For example, despite few-layer crystals being thin enough to neglect phase matching conditions, constructive and destructive interferences between surface and bulk contributions could cause dramatic deviations from the standard quadratic dependence of the SHG intensity versus crystal thickness^29^. Further, unlike simple optical interference effects that occur between reflections of the same source at different interfaces^30^, the interference of two different nonlinear mechanisms potentially provides a new handle to engineer the overall nonlinear response of a material.

In this work, we observed an unexpected peak in the SHG intensity as we varied the thickness of InSe from a few atomic layers to tens of nanometers. Our measurements exhibited a dramatic deviation from the quadratic increase in SHG expected for thin-crystals. Independent of the InSe crystal thickness, we have shown that our measured second harmonic intensity increases as the square of the off-resonant, near-infrared (NIR) fundamental pulse intensity; and exhibits a polarization dependence corresponding to the crystal symmetry of γ-InSe – a van der Waals crystal with broken-inversion symmetry for all thicknesses. InSe crystals show comparable or larger second order nonlinearities compared to GaSe, making it one of the most efficient nonlinear van der Waals crystals to date. To explain our results, we have used a simple model, taking into account complex refractive indices of the sample and substrate, the magnitude and phase of the fundamental, and the sample thickness, to calculate amplitudes and phases of reflected surface and bulk contributions, and their interference. The model is in good agreement with experimental data, showing that the unexpected peak in the SHG is the result of high-contrast interference effects of the surface and bulk contributions to the overall nonlinear signal.

To study nonlinear optical phenomena in InSe, we prepared a number of flakes by mechanical exfoliation from a γ-InSe bulk crystal synthesized according to the non-stoichiometric melt method^27^, and transferred them onto 0.5 mm, z-cut, bare quartz substrates using the viscoelastic stamping method^31^. Exfoliated flakes exhibited multiple thicknesses, as seen via optical contrast in a high-resolution microscope image. Thinner regions were similar in color to the substrate, while thicker regions exhibited vivid colors (Fig. 1a). For this study, we largely focused on the thickness of flakes, ranging from 9 to 25 nm, which were determined by the energy of the photoluminesence peaks^32^,^33^ and also confirmed by atomic force microscopy (AFM) (see Supplementary Information). Photoluminescence (PL) of the flakes ranged from 1.25 eV (994 nm) to 1.34 eV (927 nm). Lateral sizes of regions of constant thickness were typically a few microns. The ditriagonal-pyramidal C3v crystal structure of γ-InSe (Fig. 1b)^34^ is independent of thickness, and in agreement with spatially-resolved Low Energy Electron Diffraction (LEED) on exfoliated flakes (Fig. 1c). Further details about characterization of flakes and their thicknesses via PL and AFM are presented in the Supplementary Figure S1. Next we describe the experimental setup used for measurement of the SHG response in reflection as shown in the schematic (Fig. 1d).

To measure the nonlinear SHG response of these flakes, we used a 4 MHz, 45 fs high-power (650 nJ/pulse) Ti:sapphire oscillator centered at 800 nm as an input to an Optical Parametric Amplifier (OPA). The OPA generated 150 fs, few-nJ pulses tunable between 1.0–1.4 μm, allowing off-resonant excitation of InSe flakes. Pulses were focused through a 0.6 NA objective lens, providing ~1 μm spot size and 100 μW at the sample. The sample was placed on a motorized XY stage in order to exclusively excite specific constant-thickness regions within exfoliated InSe flakes. The reflected SHG was collected through the same objective, and its spectrum was measured using a dispersive spectrometer equipped with a thermoelectrically cooled CCD camera. A polarizer was placed after the sample to resolve the SHG polarization, and the sample was placed on an additional rotational stage in order to enable rotation of the sample azimuthal angle with respect to the fundamental pulse. Further details of the experimental setup are presented in the Supplementary Information. With this capability of measuring the second harmonic signal from InSe flakes with micron-scale resolution, we created a 2D spatial map of the InSe nonlinear response. We compared this map to an optical image as well as an AFM map of the same area.

## Results

Figure 2a shows an optical image of an InSe flake on quartz under white light illumination. The quartz appears as a nearly black background, while regions of different thickness show varying degrees of contrast. Thinner regions are darker shades of blue while thicker ones become lighter. The red box marks a location of 15 μm × 30 μm that was further imaged using the AFM (Fig. 2b), and the emitted SHG intensity (Fig. 2c). AFM measurements were taken in tapping mode under ambient conditions and show the thickness present within this region in false color ranging from 9–25 nm. The 2D map of SHG intensity was obtained by focusing the fundamental beam down to a 1 μm spot, from which SHG intensity was measured, and using an automated XY stage to cover the region of interest. In all three figures (Fig. 2a–c) the dotted outline (labeled as ‘Medium’) marks the region corresponding to a thickness of ~20 nm, producing the strongest SHG. In comparison, regions of 10 nm thickness (labeled as ‘thin’) and 25 nm thickness (labeled as ‘thick’) produced SHG signals five- and two-fold weaker, respectively. Thus we observed a rise and fall in SHG intensity peaked around a 20 nm thickness. In general, the intensity of the SHG signal is expected to quadratically increase with material thickness^29^ The peak in SHG intensity for an intermediate thickness provides a dramatic deviation from this standard behavior and suggests a different underlying mechanism.

**Figure 1:**
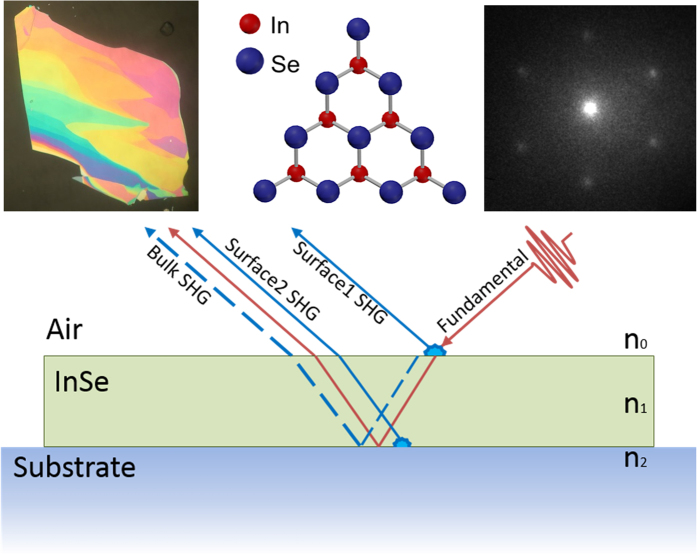
InSe crystal structure and schematic of Second Harmonic Generation geometry. (**a**) Optical microscope image showing a mechanically exfoliated InSe flake with different thicknesses displaying a variety of vivid colors. (**b**) InSe crystal structure (top view) (**c**) Low energy electron diffraction image of InSe crystal, exhibiting three-fold symmetry. (**d**) Schematic portraying the different SHG components produced in atomically thin InSe in reflection upon excitation by the fundamental beam. For certain thicknesses of InSe, SHG contributions produced due to InSe-Air and InSe-substrate interfaces are comparable to bulk contributions and produce strong interference patterns.

To further investigate the peak in the nonlinear signal at ~20 nm thickness, we next explored polarization of the SHG and its intensity versus the fundamental power for different thicknesses of InSe. For polarization measurements, the input polarization of the fundamental beam was fixed, and the sample was rotated with the beam spot as the pivot point. The polarization components of the emitted SHG (Fig. 3a) were measured parallel (black dots) and perpendicular (red dots) to the fundamental polarization as a function of the azimuthal angle of the sample – θ, and followed a cos2(3θ) (black fit) and sin2(3θ) (red fit) dependence respectively, independent of flake thickness. This six-fold symmetry of the SHG corresponds to the LEED image and the C3v crystal structure (Fig. 1b,c). Fig. 3b shows the spectrum of the generated SHG at 533 nm with a fundamental at 1066 nm with increasing fundamental intensity. We observed the expected dependence of SHG intensity as the square of the intensity of the fundamental pulse, independent of crystal thicknesses. Polarization and power dependencies of the SHG provided classic signatures for a second harmonic process for all thicknesses measured. Thus, we turned our attention to investigating the different second harmonic processes and their potential interplay that could cause an increase in the signal at specific thicknesses of InSe.

## Discussion

Recent studies have shown that monolayer GaSe exhibits very large nonlinearities for monolayer crystals, corresponding to strong surface nonlinear susceptibilities^23^. We observed comparable SHG from atomically thin layers of InSe as well (Supplementary Figure S3). At the same time, bulk second harmonic signals in 3D InSe crystals are well known^25^, and expected to grow quadratically with thickness for thin crystals. Thus, one expects an intermediate range of thicknesses in InSe, where surface and bulk contributions are comparable and can be made to interfere^35^ with different phases as a function of crystal thickness.

To account for these effects, we consider a simple model^35^,^36^ where surface nonlinear contributions at the InSe-air interface and the InSe-substrate interface interfere with the bulk nonlinear contribution that grows quadratically with the crystal thickness (Fig. 1d). The model is completely determined by two key parameters: the relative contributions of the surface and bulk nonlinear contributions; and the complex refractive index of few-layer InSe, n_InSe_. The refractive index of the quartz substrate – n_quartz_ is taken from literature^37^. We ignore phase matching for InSe thicknesses under consideration. Amplitudes and phases of the reflected SHG contributions are determined by their Fresnel coefficients. Details of the model are presented in the Supplementary Information.

**Figure 2:**
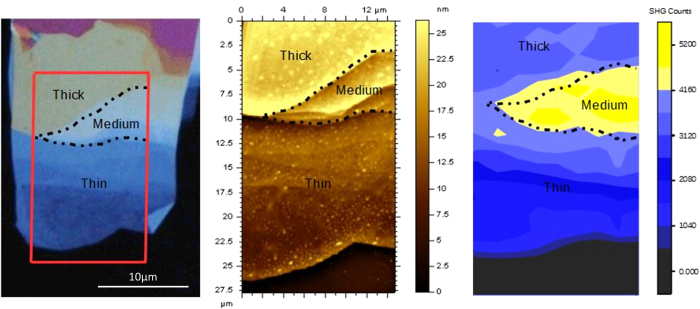
2D map of SHG generated from an InSe flake with regions of different thickness. (**a**) High resolution optical image of InSe flake. The red box shows the roughly 15 × 35 μm scan area for b and c. (**b**) Atomic Force Microscopy (AFM) image of the flake showing thicknesses ranging from 9 to 25 nm. (**c**) 2D map with ~1 μm spatial-resolution of the emitted SHG from different regions of the InSe flake. We see different strengths of SHG intensity from different thicknesses. In all three figures, the dashed black line marks the region of ~20 nm thickness (labeled as ‘Medium’) which emits a five-fold and two-fold greater SHG signal compared to the regions of 10 nm thickness (labeled as ‘Thin’) and regions of 25 nm thickness (labeled as ‘Thick’) respectively.

**Figure 3:**
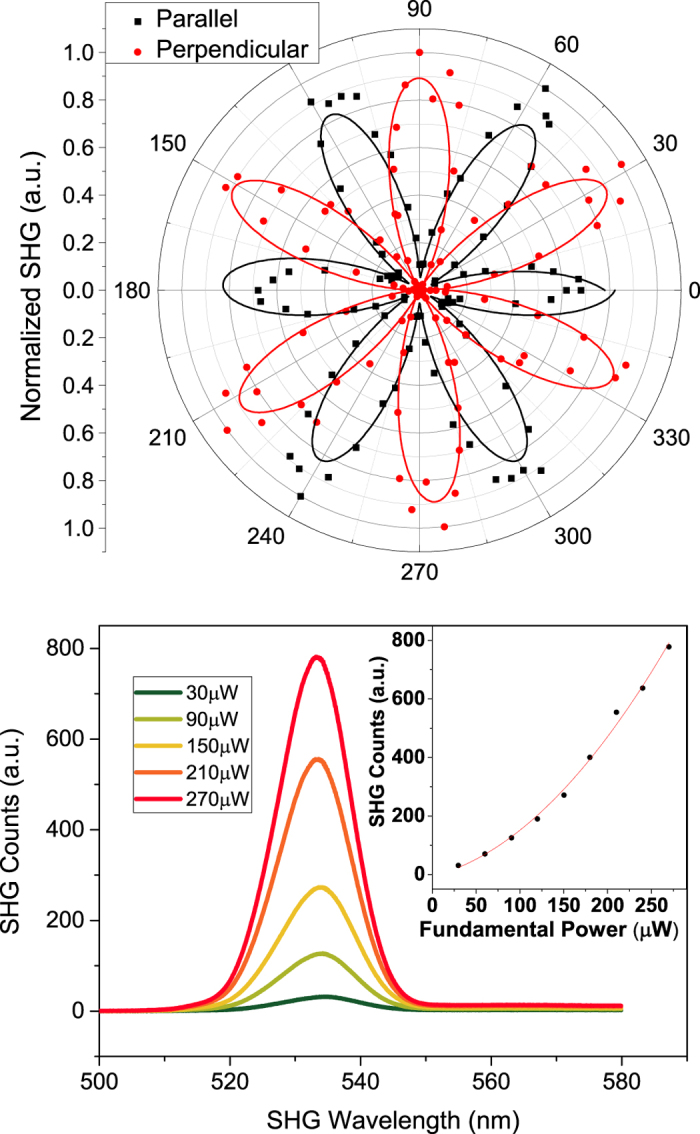
Polarization and Power dependence of SHG. (**a**) Measured intensity of SHG components perpendicular (red dots) and parallel (black dots) to the fundamental polarization as the sample is rotated. Black and red lines are their respective fits showing a cos2(3θ) and a sin2(3θ) dependence, respectively. (**b**) SHG spectrum and intensity, which scales as the square of the intensity of the fundamental beam (inset).

**Figure 4:**
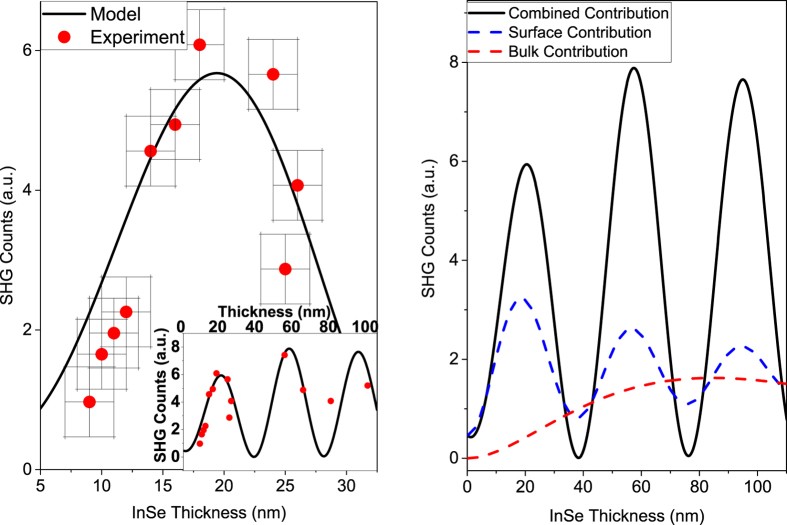
Model and experimental data for emitted SHG intensity versus InSe thickness. (**a**) Detailed plot of measured SHG counts (red dots) versus InSe thickness in the 9–25 nm range. Error bars show estimated error for thickness and SHG measurements. The unexpected peak at ~20 nm thickness in the SHG intensity is explained as constructive interference between surface and bulk components in our model (black line). Inset: SHG counts versus thickness for slightly larger thicknesses. (**b**) Surface (dashed blue) and bulk (dashed red) nonlinear contributions in our model plotted separately, which give rise to the high contrast interference of the total nonlinear response (solid black line) as a function of InSe thickness. The relatively weaker oscillations seen in the surface component (dashed blue) are a result of interferences between the contributions from the InSe-Air and the InSe-substrate interfaces. Particular to van der Waals crystals, one sees a large surface contribution even for monolayers, which can then be comparable to the bulk nonlinear contribution, as the crystal thickness can be fine-tuned layer-by-layer, thus creating a novel opportunity to study their interplay and optimize their nonlinear response.

Figure 4a shows a detailed plot of the measured SHG Intensity (red dots), for different samples than those of Fig. 2c, versus InSe crystal thicknesses in the range of 9–25 nm. We see the peak at ~20 nm corresponding to Fig. 2c, which is reproduced well by our model (black line). This peak is explained by the interference between surface and bulk contributions as seen in Fig. 4b. The surface contribution (dashed blue line) is the combined contribution due to both the Air-InSe and InSe-substrate interfaces, which also have a relative phase depending on the InSe thickness and hence show oscillations, albeit weaker, versus crystal thickness. The bulk contribution (dashed red line) increases quadratically for small thickness until absorption effects begin to impact the SHG intensity. We see that for intermediate thicknesses, the bulk contribution is comparable to the strong surface contribution, which results in high-contrast oscillations in the overall nonlinear signal.

In conclusion, we have explored the transition of nonlinear optical phenomena in going from atomically thin, 2D crystals towards larger, tens of nanometer sized 3D crystals of InSe. We showed that van der Waals crystals allow for the interplay between comparable surface and bulk nonlinear contributions, due to the large nonlinear susceptibilities of monolayers, and the ability to finely tune the bulk contribution by increasing the crystal thickness layer-by-layer. We observed interference effects between the distinct surface and bulk contributions – a clear deviation from the expected quadratic increase in the SHG signal with thickness for thin crystals. The ability to interfere two different nonlinear contributions presents interesting possibilities in the engineering of nonlinear optical phenomena. For example, by choosing different substrates or capping layers, one could potentially further manipulate the relative phases and contributions of the two nonlinearities to enhance or suppress the overall signal. The ability to perform these manipulations and enhancements in the tens of nanometer range has particular implications for the use of thin crystals for nonlinear optical devices. Overall, the observation of the interplay between surface/interface and bulk nonlinearities also raises intriguing questions regarding nonlinear phenomena in van der Waals heterostructures containing multiple ‘bulk’ regions of different nonlinear crystals, and multiple interfaces between them.

## Methods

### Sample Preparation

First, bulk InSe was synthesized by nonstoichiometric melt method as previously reported^27^. The molar ratio between indium (>99.99% Alfa Aesar Co.) and selenium (>99.99% Sigma Aldrich Co.) was 52:48. The precursor was sealed in a vacuum tube and heated to 685 °C for the reaction. Then the temperature was increased to 700 °C and maintained for 3 hours and then slowly cooled to 500 °C at a ramp rate of 10°/h, followed by natural cooling to room temperature.

From the bulk crystal, InSe flakes of varying thicknesses were prepared by mechanical exfoliation and transferred onto 0.5 mm thick z-cut quartz substrates using the viscoelastic stamping method^31^. InSe displays a 1.25 eV direct bandgap as a bulk material, but transitions to an expected 2.4 eV indirect bandgap for a single layer^32^. Thicknesses of InSe flakes were determined by the energy of the PL peak and confirmed by atomic force microscopy (AFM). Supplementary Figure S1 shows an example of two such flakes, for which PL and AFM measurements were made. Flakes with optical colors ranging in blue correspond to thinner crystals, while other dramatic colors are generally thicker crystals. Crystals that were very thin were nearly the same shade as the quartz and produced no PL, presumably because of their shift to indirect bandgap.

### Experimental Setup

Second harmonic generation (SHG) of exfoliated crystals was studied using a longpass high power Ti:sapphire oscillator system operating at 4 MHz with 650 nJ/pulse at 800 nm central wavelength and 45 fs pulses. The beam was sent through an optical parametric amplifier (see Supplementary Figure S2 online), producing a 1000–1400 nmm tunable output with a ~150 fs pulse duration at the sample. The beam was focused onto InSe flakes with a 0.60 NA objective lens to nearly 1 μm, and the reflected SHG was sent through a spectrometer and measured with a thermoelectrically cooled CCD camera. To study surface electronic polarizability, an analyzer was placed after the sample to measure only the component of the SHG coplanar or crossplanar with the fundamental pump beam. The sample was mounted on two sets of XY translation stages on a larger rotational stage. This allowed the sample to be moved to the center of rotation, such that the InSe crystal of interest could be rotated without translating. For the map of SHG, samples were placed on a motorized XY stage with submicron resolution. The sample was then scanned to collect a SHG spectrum at each point on the sample. For all SHG measurements, the fundamental exciting the sample was typically set below 0.1 mW to prevent any possible damage.

## Additional Information

**How to cite this article**: Deckoff-Jones, S. *et al.* Observing the interplay between surface and bulk optical nonlinearities in thin van der Waals crystals. *Sci. Rep.* **6**, 22620; doi: 10.1038/srep22620 (2016).

## Supplementary Material

Supplementary Information
