# Supplementary material for: Observing the interplay between surface and bulk optical nonlinearities in thin van der Waals crystals
Source: Sci Rep. 2016 Mar 3;6:22620. doi: 10.1038/srep22620 (PMC4776178; doi:10.1038/srep22620)
Supplement: Supplementary Information [file srep22620-s1.pdf]

Supplementary Information

Observing the interplay between surface and bulk optical nonlinearities in thin van der Waals crystals

Skylar Deckoff-Jones[1], Jingjing Zhang[1], Chris Petoukhoff[1], Michael K.L. Man[1], Sidong Lei[2], Robert Vajtai[2], Pulickel M. Ajayan[2], Diyar Talbayev[3], Julien Madéo[1], Keshav M. Dani[1]*

[1]Femtosecond Spectroscopy Unit, Okinawa Institute of Science and Technology Graduate University, 1919-1 Tancha, Onna, Japan.

[2]Department of Materials Science and NanoEngineering, Rice University, Houston, Texas, 77005, USA

[3]Department of Physics and Engineering Physics, Tulane University, 6400 Freret Street, New Orleans, Louisiana 70118, US

Section 1: Sample Preparation

First, bulk InSe was synthesized by nonstoichiometric melt method as previously reported.[1] The molar ratio between indium (>99.99% Alfa Aesar Co.) and selenium (>99.99% Sigma Aldrich Co.) were 52:48. The precursor was sealed under vacuum tube and heated to 685 degrees Celsius for reaction, then the temperature was increased to 700 degrees Celsius and maintained for 3 hours and then slowly cooled to 500 degrees Celsius at a ramp rate of 10 degrees per hour, followed by natural cooling to room temperature.

From the bulk crystal, InSe flakes of varying thicknesses were prepared by mechanical exfoliation transferred onto 0.5mm thick z-cut quartz substrate using the viscoelastic stamping method.[2] InSe displays a 1.25eV direct bandgap as a bulk material but transitions to an expected 2.4eV indirect bandgap for a single layer.[3]

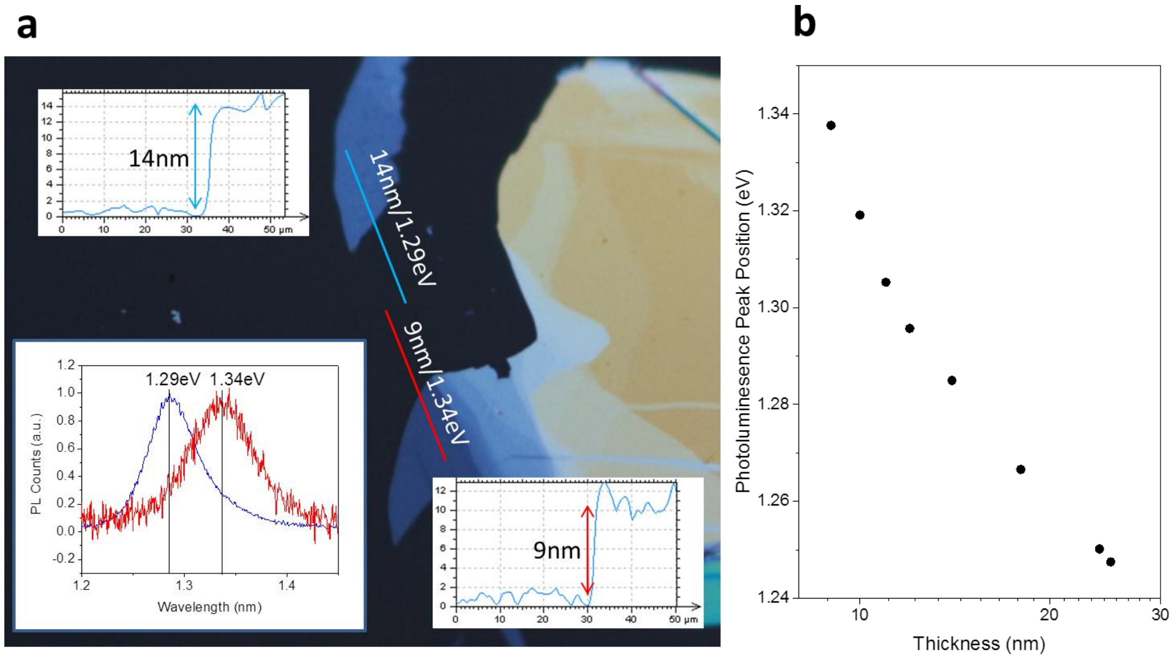

Supplementary Figure S1| Background image of two of the measured flakes with their AFM and PL measurements (a). The same measurements of PL and AFM were made for 8 different InSe crystals and plotted in b which is in agreement with.3

The thickness of the InSe flakes were determined by the energy of the photoluminesence peak and confirmed by atomic force microscopy (AFM). Supplementary Figure S.1a shows an example of two such flakes, for which PL and AFM measurements were made. Supplementary Figure S.1b presents the measured PL for flakes with thicknesses varying from 9 to 25 nm. Flakes with optical colors ranging in blue correspond to thinner crystals, while the other dramatic colors are generally thicker crystals. Crystals that were very thin were nearly the same shade as the quartz and produced no PL, presumably because of their shift to indirect bandgap.

Section 2: Experimental Setup

The exfoliated crystal's second harmonic generation (SHG) was studied using a longpass high power Ti:sapphire oscillator system operating at 4MHz with 650nJ/pulse at 800nm central wavelength and 45 fs pulses. The beam was sent through an optical parametric amplifier, producing a 1000-1400nmm tunable output with ~150 fs pulse duration at the sample. The beam was focused onto the InSe flakes by a 0.60 NA objective lens to nearly 1 µm, and the reflected SHG was sent through a spectrometer and measured with a thermoelectrically cooled CCD camera. To study the surface electronic polarizability, an analyzer was placed after the sample to measure only the component of the SHG coplanar or crossplanar with the fundamental pump beam. The sample was mounted on two sets of XY translation stages on a larger rotational stage. This allowed the sample to be moved to the center of rotation, such that the InSe crystal of interest could be rotated without translating. For the map of SHG, the samples were placed on a motorized XY stage with submicron resolution. The sample was then scanned to collect a SHG spectrum at each point on the sample. For all SHG measurements, the fundamental exciting the sample was set below typically 0.1 mW to prevent any possible damage.

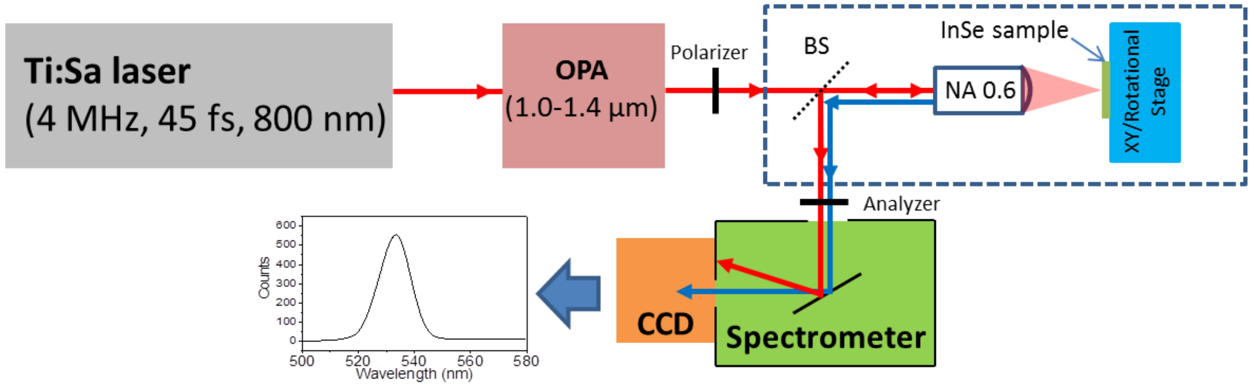

Supplementary Figure S2| Diagram of experimental setup.

Section 3: Comparison of the InSe and GaSe Nonlinear Response

InSe and GaSe flakes of similar thickness (~20nm) were identified optically and measured by AFM. The SHG under the same condition on z-cut quartz substrate was measured. The results show InSe to show greater SHG by nearly a factor of two.

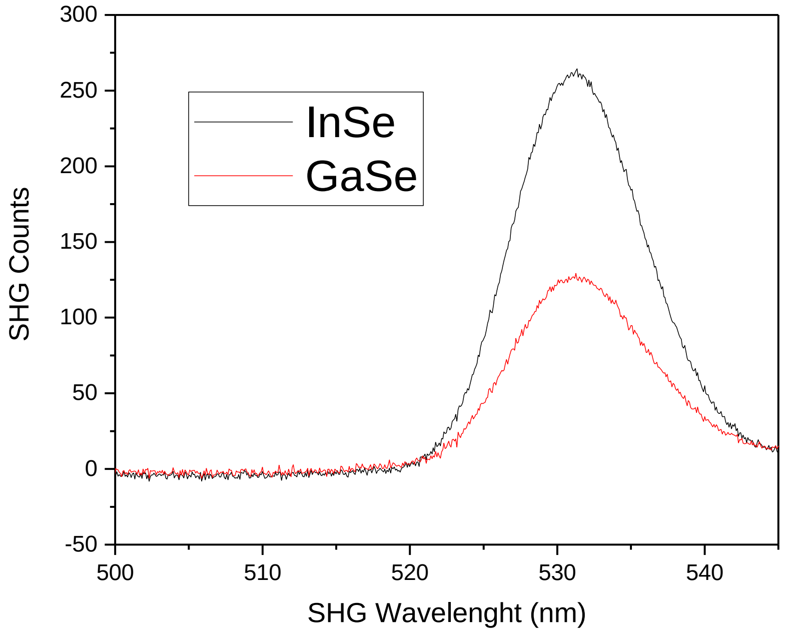

Supplementary Figure S3| SHG of GaSe, red, and InSe, black, under similar conditions.

Section 4: Model

We describe in this section the model used to reproduce the experimental data. This model accounts for interference between surface and bulk SHG without considering phase mismatch from the bulk contribution. We consider a geometry composed of three layers: air (layer 0), Inse (layer 1) and the quartz substrate (layer 2) for which the refractive indexes are $n_0, \tilde{n}_1(\omega) = n_1(\omega) + in'_1(\omega)$ and n_2 where only the index $\tilde{n}_1(2\omega)$ of the InSe layer is assumed to have a non-zero imaginary part as the SH wavelength is above the bandgap and thus absorbed. The total SH intensity as a function of the sample thickness can be written as:[4, 5]

$$I_{SHG}(L) = \frac{32\pi^3\omega^2}{c^3 n_1(\omega) n_1(2\omega)^2} |S(L) + B(L)|^2 {I_0}^2 \qquad \text{Supplementary Equation S1}$$

where L is the InSe thickness, S the contribution from the top (air/InSe) and bottom (InSe/quartz) surfaces and B the contribution from the bulk. For simplicity, only the two first contributions of the multiple reflections are considered in the calculation of S and B. Thus, we can write:

$$S(L) = \chi_s^{(2)}\left(R_{01} + T_{01}T_{10}R_{12}e^{2iKL} + {t_{10}}^2 R_{12}T_{10}e^{2ikL}e^{iKL}\right) \qquad \text{Supplementary Equation S2}$$

and

$$B(L) = \chi_b^{(2)} L\left(T_{10}{t_{10}}^2 R_{12}e^{2iKL} + {t_{10}}^2 {r_{12}}^2 T_{10}e^{2ikL}e^{iKL}\right) \qquad \text{Supplementary Equation S3}$$

where $r_{ij} = \frac{n_i - n_j}{n_i + n_j}$, $t_{ij} = \frac{2n_i}{n_i + n_j}$ are the Fresnel coefficients at the fundamental wavelength λ_ω and $R_{ij} = \frac{n_i - n_j}{n_i + n_j}$, $T_{ij} = \frac{2n_i}{n_i + n_j}$ at the second harmonic wavelength $\lambda_{2\omega}$. $k = \frac{2\pi\tilde{n}_1(\omega)}{\lambda_\omega}$ and $K = \frac{2\pi\tilde{n}_1(2\omega)}{\lambda_{2\omega}}$ are the wavevectors at the fundamental and SH wavelengths, respectively. $\chi_s^{(2)}$ and $\chi_b^{(2)}$ are second order nonlinear susceptibilities for the InSe surfaces and bulk, respectively. The linear dependence with L in Supplementary Equation S3 accounts for the SHG as the fundamental propagates through the InSe.

The parameters used to reproduce the experimental data are: $n_0 = 1$, $\tilde{n}_1(\omega) = 7$, $\tilde{n}_1(2\omega) = 7 + i0.5$, $n_2 = 1.46$. The high value for the real part of the InSe refractive index is in agreement with recent results showing high refractive indices for optically-active atomically thin van der Waals crystals.[6, 7] $\frac{\chi_s^{(2)}}{\chi_b^{(2)} L}$ lies between 0.05 and 0.55 corresponding to a ratio of the bulk to surface contribution, $\left|\frac{B(L)}{S(L)}\right|^2$,ranging from 0.1 to 1 for L varied between 9nm to 100nm.

REFERENCES

1. Lei, S.; Ge, L.; Najmaei, S.; George, A.; Kappera, R.; Lou, J.; Chhowalla, M.; Yamaguchi, H.; Gupta, G.; Vajtai, R.; Mohite, A. D.; Ajayan, P. M. *ACS Nano* 2014, 8, (2), 1263-72.

2. Castellanos-Gomez, A.; Buscema, M.; Molenaar, R.; Singh, V.; Janssen, L.; van der Zant, H. S. J.; Steele, G. A. *2D Materials* 2014, 1, (1), 011002.

3. Mudd, G. W.; Svatek, S. A.; Ren, T.; Patane, A.; Makarovsky, O.; Eaves, L.; Beton, P. H.; Kovalyuk, Z. D.; Lashkarev, G. V.; Kudrynskyi, Z. R.; Dmitriev, A. I. *Adv Mater* 2013, 25, (40), 5714-8.

4. Mizrahi, V.; Sipe, J. E. *Journal of the Optical Society of America B* 1988, 5, (3), 660.

5. Koopmans, B.; Anema, A.; Jonkman, H. T.; Sawatzky, G. A.; van der Woude, F. *Physical Review B* 1993, 48, (4), 2759-2764.

6. Yang, J.; Wang, Z.; Wang, F.; Xu, R.; Tao, J.; Zhang, S.; Qin, Q.; Luther-Davies, B.; Jagadish, C.; Yu, Z.; Lu, Y., Atomically Thin Optical Lenses and Gratings. In *ArXiv e-prints*, 2014; Vol. 1411, p 6200.

7. Castellanos-Gomez, A.; Agraït, N.; Rubio-Bollinger, G. *Applied Physics Letters* 2010, 96, (21), 213116.
